# Asthmatic Eosinophils Promote Contractility and Migration of Airway Smooth Muscle Cells and Pulmonary Fibroblasts In Vitro

**DOI:** 10.3390/cells10061389

**Published:** 2021-06-04

**Authors:** Ieva Janulaityte, Andrius Januskevicius, Virginija Kalinauskaite-Zukauske, Jolita Palacionyte, Kestutis Malakauskas

**Affiliations:** 1 Laboratory of Pulmonology, Department of Pulmonology, Lithuanian University of Health Sciences, LT-44307 Kaunas, Lithuania; andrius.januskevicius@lsmuni.lt (A.J.); kestutis.malakauskas@lsmuni.lt (K.M.); 2 Department of Pulmonology, Lithuanian University of Health Sciences, LT-44307 Kaunas, Lithuania; virginija.kalinauskaite@lsmuni.lt (V.K.-Z.); jolita.palacionyte@lsmuni.lt (J.P.)

**Keywords:** asthma, pathogenesis, eosinophil, airway smooth muscle cell, pulmonary fibroblast, migration, contractility, extracellular matrix

## Abstract

Enhanced contractility and migration of airway smooth muscle cells (ASMC) and pulmonary fibroblasts (PF) are part of airway remodeling in asthma. Eosinophils are the central inflammatory cells that participate in airway inflammation. However, the role of asthmatic eosinophils in ASMC and PF contractility, migration, and differentiation to contractile phenotype has not yet been precisely described. A total of 38 individuals were included in this study: 13 steroid-free non-severe allergic asthma (AA) patients, 11 severe non-allergic eosinophilic asthma (SNEA) patients, and 14 healthy subjects (HS). For AA patients and HS groups, a bronchial allergen challenge with *D. pteronyssinus* was performed. Individual combined cell cultures were prepared from isolated peripheral blood eosinophils and immortalized ASMC or commercial PF cell lines separately. The migration of ASMC and PF was evaluated using wound healing assay and contractility using collagen gel assay. Gene expression of contractile apparatus proteins, COL1A1, COL5A1, and FN, in ASMC and PF was evaluated using qRT-PCR. We found that contractility and migration of ASMC and PF significantly increased after incubation with asthmatic eosinophils compared to HS eosinophils, *p* < 0.05, and SNEA eosinophils demonstrated the highest effect on contractility of ASMC and migration of both cell lines, *p* < 0.05. AA and SNEA eosinophils significantly increased gene expression of contractile apparatus proteins, COL1A1 and FN, in both cell lines, *p* < 0.05. Furthermore, the allergen-activated AA eosinophils significantly increased the contractility of ASMC, and migration and gene expression in ASMC and PF, *p* < 0.05. Thus, asthmatic eosinophils change ASMC and PF behavior by increasing their contractility and migration, contributing to airway remodeling.

## 1. Introduction

In recent years, growing numbers of asthma patients and uncontrolled episodes indicate the need for fundamental asthma pathogenesis studies [1]. Airway remodeling is a crucial feature of asthma pathogenesis. The thickened airway wall is composed of structural cells, deposited extracellular matrix (ECM) proteins, and migrated inflammatory cells. Structural lung cells, such as airway smooth muscle cells (ASMC) and pulmonary fibroblasts (PF), are the synthetically and mechanically active cells that quickly respond to airway inflammation by changing their behavior via the release of various biologically active mediators, production of ECM proteins, and increased contraction and migration.

Altered contractility and migration of ASMC and PF are part of airway inflammatory processes that contribute to airway remodeling in asthma. Previous studies have shown that ASMC from asthma patients are different from non-asthmatic subjects, being hypercontractile, hyperproliferative, and hypersecretory [2,3,4]. In asthma, ASMC are thought to generate more force and, therefore, contract to a greater extent or to have increased maximum shortening velocity and capacity [5,6]. Allergic sensitization of human airways results in increased levels of myosin light chain kinase (MLCK), which phosphorylates the myosin light chain (MHC) and leads to ASMC contraction [7]. The dynamics of ASMC contractility and relaxation in asthma are discussed to have a key role in airway narrowing [8]. Contractility and migration of ASMC are closely related because migration requires highly conserved molecular machinery to coordinate the contraction [9]. The increased ASMC mass in asthmatic airways due to increased migration and proliferation is one of the airway remodeling features.

PF are the main cells that are responsible for ECM proteins homeostasis in airways. Furthermore, the PF migrate to the inflammation site and are the main cells responsible for wound healing. It was shown that migrated PF differentiate into myofibroblasts that contract and excessively produce ECM proteins in asthma, resulting in subepithelial fibrosis [10]. Myofibroblasts are also characterized by increased focal adhesions, cell-to-cell junctions, and *α*-sm-actin expression [11]. In addition, ASMC and PF secrete myriad growth factors and cytokines that promote airway inflammation [12,13,14]. Thus, ASMC and PF have a critical role in airway remodeling in asthma.

Eosinophils migrated to the lungs adhere to structural cells and release transforming growth factor *β* (TGF-*β*), reactive oxygen species, eosinophil-derived neurotoxin, eosinophil peroxidase, major basic protein, eosinophil cationic protein, and other biologically active mediators that promote inflammation and destruction of neighboring cells [15]. Structural cells become activated under these conditions, resulting in differentiation of pathological phenotypes—ASMC can differentiate into more active proliferative-synthetic or contractile phenotypes and PF into myofibroblasts. Airway remodeling processes induced by eosinophils are the basis of asthma pathogenesis.

The novelty of our study is that we used eosinophils isolated from the blood of patients with different severeness of asthma and used them in combined cultures with ASMC and PF cell lines showing the different eosinophil effects on their contractility and migration. This study model imitates in vivo processes. We hypothesized that blood eosinophils are activated in the bone marrow, thus representing an ability to participate in airway remodeling processes and might be involved in ASMC and PF differentiation into more active contractile phenotypes. So, this study aimed to estimate the differences between the effects of asthmatic and healthy eosinophils on structural lung cell contractility and migration.

## 2. Materials and Methods

The research protocol was approved by the Kaunas Regional Biomedical Research Ethics Committee of the Lithuanian University of Health Sciences with permission no. BE-2-13. The research study was registered in the US National Institutes of Health trial registry ClinicalTrials.gov with identifier NCT03388359.

### 2.1. Study Subjects

The study group consisted of 13 allergic asthma (AA) patients, 11 severe non-allergic eosinophilic asthma (SNEA) patients, and 14 healthy subjects (HS) aged between 18 and 80 years. SNEA and AA patients were recruited from the Department of Pulmonology at the Hospital of Lithuanian University of Health Sciences Kauno klinikos. All study participants gave written informed consent, and in the recruitment stage, all subjects were screened: they underwent clinical examination, spirometry, methacholine challenge test, skin prick test, and complete blood count analysis.

The applied inclusion and exclusion criteria for all groups are presented in Table 1.

The inclusion and exclusion criteria were checked at the screening visit, and the study subjects signed informed consent. Then, spirometry was performed on all study groups. For AA and HS study groups, the methacholine challenge test and skin prick test was performed. In addition, during the baseline visit, the blood samples were collected, and bronchial allergen challenge with *D. pteronyssinus* was performed for AA and HS study groups. Twenty-four hours after the bronchial allergen challenge, the second study visit was scheduled for AA patients and HS, and blood samples were re-taken. For SNEA patients, only one visit was scheduled during which the blood samples were collected.

The eosinophils were isolated from subjects’ peripheral blood samples using high-density centrifugation and magnetic separation. We used subjects’ eosinophils and evaluated migration of ASMC and PF using a wound-healing assay, and measured contractility of ASMC and PF using collagen gel assay and gene expression of the contractility markers and ECM proteins in both cell lines.

**Figure 1:**
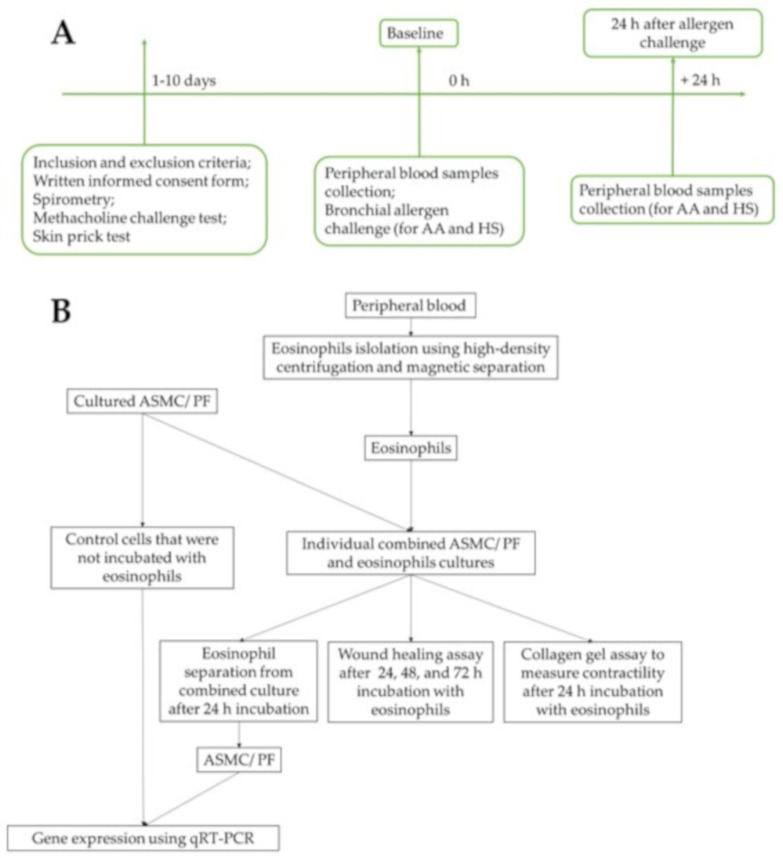
Flowchart of the study design. (**A**)—Recruitment of study subjects, clinical examination of allergic asthma patients and healthy subjects. (**B**)—Experimental workflow. AA—allergic asthma; ASMC—airway smooth muscle cells; HS—healthy subjects; PF—pulmonary fibroblasts; qRT-PCR—quantitative reverse transcription polymerase chain reaction.

**Figure 2:**
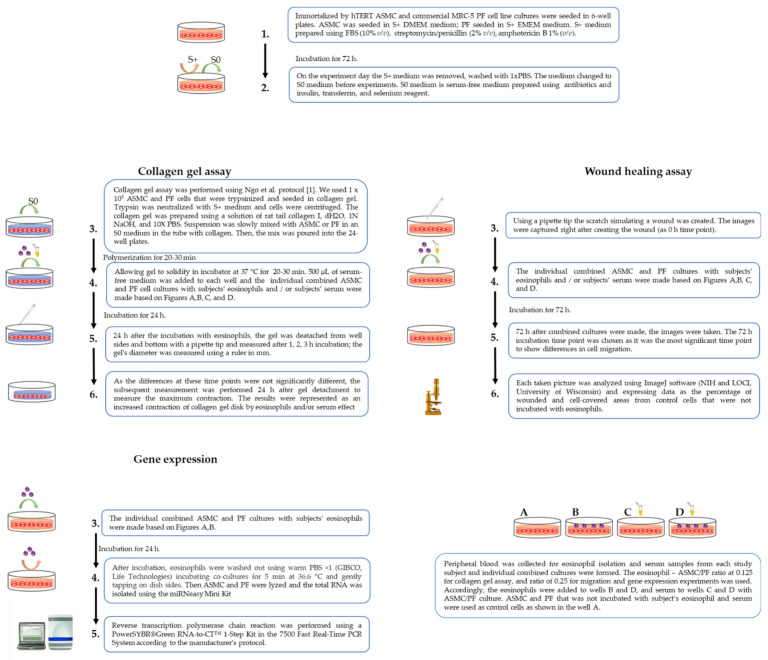
The detailed experimental plan. ASMC—airway smooth muscle cells; PF—pulmonary fibroblast.

A flow chart of the study design and experimental workflow is presented in Figure 1. The detailed experimental plan is provided in Figure 2.

### 2.2. Lung Function Testing

The lung function of study subjects was evaluated according to baseline forced expiratory volume in 1 s (FEV_1_), forced vital capacity (FVC), and FEV1/FVC ratio using a Ganshorn spirometer (Ganshorn Medizin Electronic, Niederlauer, Germany). Baseline FEV1, FVC, and FEV1/FVC ratios were recorded as the highest result of three reproducible measurements compared to the predicted values matched for body height, weight, age, and sex using standardized methodology. Each of the values was repeatedly measured at least three times that met standards, but no more than eight times, and the highest value of FEV1 was taken for analysis.

### 2.3. Measurement of Airway Responsiveness to Methacholine

AA and HS study group subjects underwent measurement of airway responsiveness to methacholine. For airway responsiveness evaluation, the inhaled methacholine test was performed using a ProvoX pressure dosimeter (Ganshorn Medizin Electronic). Aerosolized methacholine was inhaled at 2 min intervals, with a starting dose of 0.0101 mg. Then, the dose was increased by steps up to 0.121, 0.511, and 1.31 mg cumulative dose until the total cumulative dose was achieved or received the 20% decrease in FEV_1_ from the baseline. The provocative methacholine dose causing a ≥20% fall in FEV1 (PD_20M_) was calculated using the logarithmic dose-response curve by linear interpolation of the two adjacent data points.

### 2.4. Skin Prick Test

The skin prick test was conducted using standardized allergen extracts from (Stallergenes S.A., Antony, France) for the following allergens: *D. pteronyssinus*, *D. farinae*, birch pollen, and five mixed grass pollens. The histamine hydrochloride (10 mg/mL) was used as a positive control, and the negative control was diluent (saline). The skin prick test was evaluated after 15 min of application. The test results were considered positive if the wheel diameter was at least 3 mm. Only AA patients sensitized to *D. pteronyssinus* were included in the study.

### 2.5. Bronchial Allergen Challenge

All study subjects from AA and HS groups underwent bronchial allergen challenge with *D. pteronyssinus* allergen (Stallergenes S.A.). The broncho-constricting effect of nebulized saline was first assessed. The aerosolized allergen was inhaled at 10 min intervals starting with 0.1 histamine equivalent prick (HEP)/mL allergen concentration, increasing it sequentially to 1.0, 10.0, 20.0, 40.0, 60.0 HEP/mL or until a 20% decrease in FEV1 from the baseline was achieved. The allergen’s provocative dose was calculated from the log dose-response curve by the linear interpolation of two adjacent data points.

### 2.6. Isolation of Eosinophils from Peripheral Blood

Peripheral blood from each study subject was collected in vacutainers with dipotassium ethylenediaminetetraacetic acid (K2EDTA) (BD Vacutainer^®^, Becton Dickinson UK Ltd., Wokingham, UK) before and 24 h after bronchial allergen challenge from AA and HS, and at the baseline visit from SNEA patients. A UniCel^®^ DxH 800 Coulter^®^ Cellular Analysis System automated hematology analyzer (Beckman Coulter, Miami, FL, USA) was used for the complete blood count test. Whole eosinophils’ isolation from peripheral blood procedure is described in our previous publication [16].

### 2.7. Combined Cell Culture of Eosinophils and ASMC or PF

Individual combined cell cultures of eosinophils and ASMC or PF were prepared for experiments. ASMC are immortalized by stable expression of human telomerase reverse transcriptase (hTERT), as Gosens et al. described [17], and the commercial MRC-5 cell line (Sigma, Ronkonkoma, NY, USA) as PF were used. ASMC and PF were grown to 90–95% confluence in medium supplemented with 10% FBS for 72 h. Then, cells were serum-deprived before experiments, ensuring that the cells were in the phase of growth arrest, thereby equalizing all cells into the same phase of the cell cycle and minimizing the possible influence of proliferation. Isolated eosinophils were used to make the combined cultures with ASMC or PF.

For collagen gel assay, the 1.25 × 10^4^ eosinophil suspension was added to ASMC or PF that were grown in 24 well plates at each seeding at 1 × 10^5^ cell confluency. For wound healing assay, ASMC and PF were grown in 6 well plates at each seeding at 2 × 10^5^ ASMC or PF; after 72 h, the combined cultures were formed with isolated eosinophils by adding 5 × 10^4^ of them to each well. For gene expression, the ASMC and PF were cultivated in dishes with approximately 2 × 10^5^ cells, and combined cultures were made by adding 5 × 10^4^ isolated viable eosinophils’ suspension in the medium of the ASMC or PF. Each experiment was normalized using the control ASMC and PF cell culture that was not incubated with eosinophils. An inverted microscope (CETI Inverso TC100, Medline Scientific, Oxford, UK) was used for cell growth observation and visualization.

### 2.8. Culture Medium Treatment with Serum

Serum from each investigated study subject was collected into BD Vacutainer^TM^ SST^TM^ II Advance tubes (BD Vacutainer^®^, Becton Dickinson UK Ltd.) and centrifugated at 2000× *g* for 10 min. According to the growth medium supplements, ASMC and PF collagen gel assay and wound healing assay experiments were divided into the following experiments on the study day: the first part of the experiments was made with serum-free medium using only ASMC or PF cells as control cells; the second part used serum-free individual combined cultures with ASMC or PF cells and eosinophils; the third part used individual ASMC or PF cells with subject’s serum at a concentration of 2% *v*/*v*; and the fourth part used individual combined cultures of ASMC or PF cells and eosinophils supplemented with subject’s serum at a concentration of 2% *v*/*v* (Figure 2A–D). Experiments using serum were conducted to maintain further eosinophil activation after isolation processes and to verify if the eosinophils were isolated in their activated form.

### 2.9. Collagen Gel Assay

**Figure 3:**
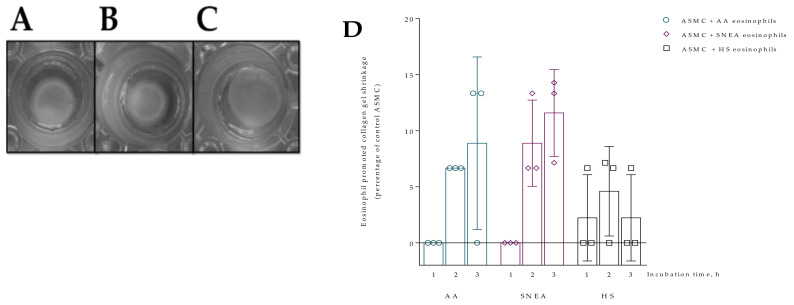

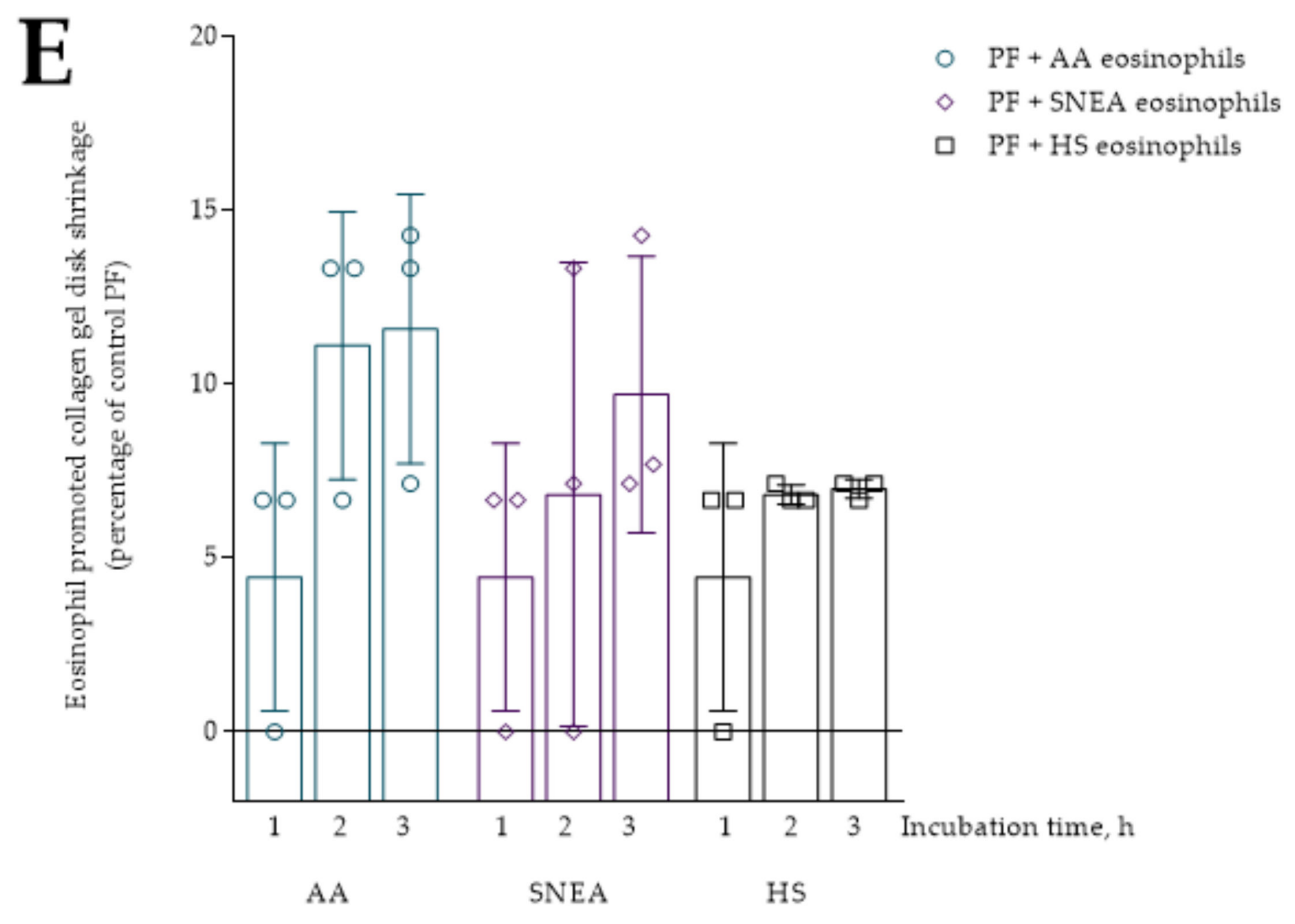
Collagen gel assay. (**A**)—ASMC after 24 h incubation with HS eosinophils; (**B**)—ASMC after 24 h incubation with SNEA patients’ eosinophils; (**C**)—ASMC after 24 h incubation with AA patients’ eosinophils; (**D**)—collagen gel contraction percentage from control ASMC after 1, 2, and 3 h incubation with subjects’ eosinophils, AA *n* = 3, SNEA *n* = 3, HS *n* = 3; (**E**)—collagen gel contraction percentage from control PF cells after 1, 2, and 3 h incubation with subjects’ eosinophils, AA *n* = 3, SNEA *n* = 3, HS *n* = 3. AA—allergic asthma patient; ASMC—airway smooth muscle cells; HS—healthy subject; PF—pulmonary fibroblast; SNEA—severe non-allergic eosinophilic asthma patients. Data presented as mean ± SEM.

Collagen gel assay was used to evaluate the eosinophil effect on ASMC- or PF-induced collagen gel contraction. We used the protocol of Ngo et al. to perform experiments [18]. Cells were grown for 72 h, and then the growth medium was changed to serum-free before the experiment. A solution of rat tail collagen I (Gibco™, Life Technologies, Carlsbad, CA, USA) was prepared using the manufacturer’s protocol. In a sterile tube, the dH_2_O, 1N NaOH, and 10× PBS were mixed with ASMC or PF cells in an S0 medium, and the collagen was slowly pipetted into the tube and mixed well. Then, the mix was poured into the 24 well plates. After the isolation of eosinophils, the S0 medium was added to the individual subjects’ serum in the wells. Twenty-four hours after the incubation with eosinophils, the gel was detached from the well sides and bottom with a pipette tip and measured after 1, 2, 3 h incubation; the gel’s diameter was measured using a ruler in mm (Figure 3). As the differences at these time points were not significantly different, the subsequent measurement was performed 24 h after gel detachment to measure the maximum contraction. The contractility was represented as a contraction of collagen gel disk in the percentage of control cells. All incubations were under standard culture conditions of 5% CO_2_ in air at 37 °C.

In cell-induced collagen gel contraction assay, the measurement of collagen gel with control cells was equated to 100% and represented as an increased contraction of collagen gel disk caused by eosinophils and/or serum effect using the following formula:

$$\%\;\mathrm{of}\;\mathrm{control}\;\mathrm{cell}\;\mathrm{gel}\;\mathrm{diameter}\;=\;100-\frac{\mathrm{gel}\;diameter\;24\;h\;\mathrm{after}\;\mathrm{incubation}\;\mathrm{with}\;\mathrm{eosinophils}\;\mathrm{and}/\mathrm{or}\;\mathrm{serum}\;\mathrm{in}\;\mathrm{mm}\;\times 100}{\mathrm{gel}\;\mathrm{diameter}\;24\;h\;\mathrm{after}\;\mathrm{incubation}\;\mathrm{with}\;\mathrm{control}\;\mathrm{cells}\;\mathrm{in}\;\mathrm{mm}}$$

### 2.10. Wound Healing Assay

**Figure 4:**
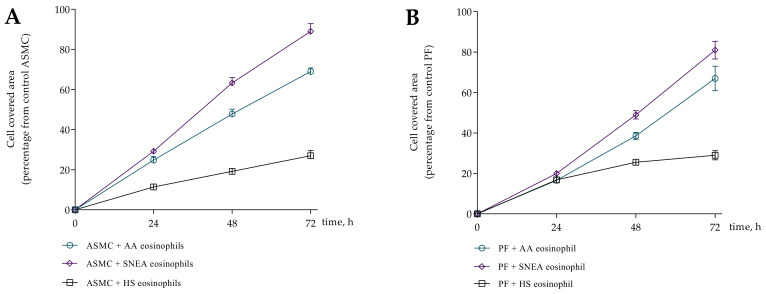
Wound healing assay. (**A**)—ASMC migration after 0 h, 24 h, 48 h, 72 h incubation with eosinophils; (**B**)—PF migration after 0 h, 24 h, 48 h, 72 h incubation with eosinophils. AA *n* = 5, SNEA *n* = 5, HS *n* = 5. Presented as a cell-covered area, as a percentage of control cells. AA—allergic asthma; ASMC—airway smooth muscle cells; HS—healthy subject; PF—pulmonary fibroblast; SNEA—severe non-allergic eosinophilic asthma patients. Data presented as mean ± SEM.

Migration of ASMC and PF cells was evaluated using wound healing assay according to Liang et al. [19]. Additionally, the subject’s serum experiments were performed by adding 20 uL of the subject’s serum. To mimic cell migration during wound healing in vivo, the wounded monolayer was co-cultured with eosinophils and/or subjects’ serum, and images were captured immediately after creating the wound (at the 0 h time point) and after 24, 48, and 72 h of incubation. Each picture taken was analyzed using ImageJ software (NIH and LOCI, University of Wisconsin) and data were expressed as the percentage of wounded and cell-covered areas from control cells that were not incubated with eosinophils (Figure 4).

First, we evaluated five AA patients, five SNEA patients, and five HS eosinophils to assess the effect on cell migration intensity at each time point; the 72 h incubation time point was chosen because it was sufficient to show differences in cell migration. The migration data is represented as the increased cell-covered area as a percentage of control cells. Images of wound healing are presented in Figure S1.

### 2.11. RNA Isolation and Quantitative Real-Time PCR Analysis

The eosinophils were separated from ASMC and PF cells after 24 h of incubation for gene expression analysis. ASMC and PF cells were lysed using TRIzol™ Reagent (Invitrogen™, Life Technologies), and the total ribonucleic acid (RNA) was isolated using the miRNeasy Mini Kit (Qiagen, Valencia, CA, USA) according to the manufacturer’s instructions. Reverse transcription polymerase chain reaction (RT-PCR) was performed using a PowerSYBR^®^Green RNA-to-CT™ 1-Step Kit (Applied Biosystems, Foster City, CA, USA) in the 7500 Fast Real-Time PCR System according to the manufacturer’s protocol. AA and SNEA eosinophils’ effects on gene expression in ASMC and PF cells were evaluated as folds over the HS eosinophil effect. Regarding the bronchial allergen effect, the gene expression changes were evaluated by folds from baseline (before allergen challenge) results. Primers used in gene expression analysis are shown in Table 2.

### 2.12. Statistical Analysis

Statistical analysis was performed using GraphPad Prism 8 for Windows (Version 8.01, 2019; GraphPad Software, Inc., San Diego, CA, USA). The Shapiro–Wilk test was used to confirm the normality assumption of data distribution. Contractility and gene expression data were not distributed normally; while migration data were distributed normally. However, the non-parametric tests were used because of a small sample sizes. For multiple comparison analysis between eosinophil effect of the investigated groups on ASMC and PF contractility and migration, the Kruskal- Wallis test was used. If Kruskal-Wallis test was significant, the Mann-Whitney two-sided *U* test was used to emphasize the different effect of eosinophil on ASMC and PF contractility and migration. For the multiple comparison within the group, the Friedman test was performed. If Friedman test was significant, the Wilcoxon matched-pairs signed-rank test was performed to define differences between eosinophil and/or serum effect on ASMC and PF contractility and migration data. Multiple comparison values are presented in the legends of Figures. Also, the Wilcoxon matched-pairs signed-rank test was used for analysis between two dependent groups to compare the data of experiments before and after bronchial allergen challenge. The Wilcoxon signed-rank test was used for gene expression analysis against the control of ASMC or PF cells. Data are presented as the mean and standard error of the mean (SEM) or standard deviation (SD). A value of *p* < 0.05 was considered statistically significant.

## 3. Results

### 3.1. Demographic and Clinical Characteristics of the Study Population

We investigated 38 nonsmoking adults (20 women and 18 men): 13 steroid-free non-severe allergic asthma (AA) patients, 11 severe non-allergic eosinophilic asthma (SNEA) patients with high doses of inhaled steroids, and 14 healthy subjects (HS). The demographic and clinical characteristics of the study population are presented in Table 3. The demographic and clinical characteristics of the study population are presented in Table 3. The SNEA patients were significantly older compared to the AA and HS groups. Furthermore, the lung function in the SNEA group was significantly lower, and the blood eosinophil count was significantly higher compared to other groups. The average BMI of SNEA patients slightly exceeded the normal BMI limit but did not differ significantly from AA and HS subjects.

The bronchial allergen challenge with *D. pteronyssinus* allergen was performed for all AA patients and the HS group (Table 4). A significant increase in the eosinophil count and immunoglobulin E (IgE) levels was observed in the blood in the AA group following allergen exposure. There were no significant changes in the HS group.

### 3.2. Contraction of Collagen Gel Disk after Incubation with Eosinophils

**Figure 5:**
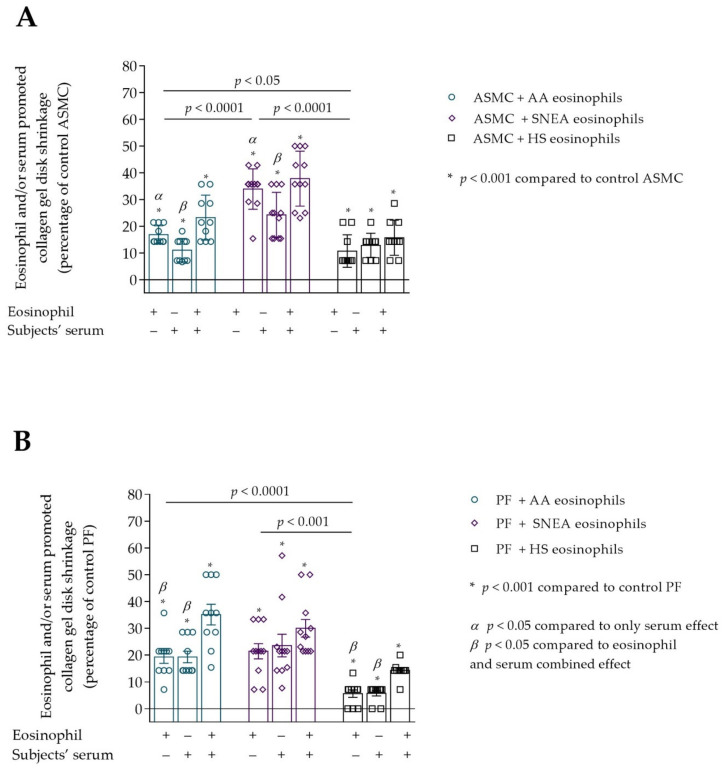
(**A**)—The effect of subjects’ eosinophils and/or serum on the ASMC-induced collagen gel disk shrinkage; (**B**)—The effect of subjects’ eosinophils and/or serum on the PF-induced collagen gel disk shrinkage. Data presented as percentage of control cells that were not incubated with eosinophils, as mean ± SEM. Added blood serum of each investigated subject to individual combined cultures: 2% *v*/*v*. AA—allergic asthma; ASMC—airway smooth muscle cells; HS—healthy subject; PF—pulmonary fibroblast; SNEA—severe non-allergic eosinophilic asthma. *α p* < 0.05 compared to the only serum effect; *β p* < 0.05 compared to eosinophil and serum combined effect. A—* *p* < 0.001 compared to control ASMC; B—* *p* < 0.01 compared to control PF. AA *n* = 12; SNEA *n* = 11; HS *n* = 10. Statistical analysis between investigated groups—Kruskal-Wallis test and post hoc Mann–Whitney two-sided *U*-test (independent data); within one study group—Friedman test for multiple comparison within the group and the post hoc Wilcoxon matched-pairs signed-rank two-sided test (dependent data). Significant differences were found in the eosinophil effect on ASMC and PF–induced collagen gel shrinkage between investigated groups by Kruskal-Wallis test, respectively, χ^2^ = 21.49, df = 2, *p* < 0.0001 and χ^2^ = 16.55, df = 2, *p* = 0.0003. Significant differences of ASMC-induced collagen gel shrinkage were found in AA and SNEA groups by Friedman test, respectively χ^2^ = 15.24, df = 2, *p* < 0.0001; χ^2^ = 11.89, df = 2, *p* = 0.0011. Significant differences of PF-induced collagen gel shrinkage were found in AA and HS groups by Friedman test, respectively, χ^2^ = 12.51, df = 2, *p* = 0.0007; and χ^2^ = 16.76; df = 2, *p* = 0.0002. Lines connect comparison groups with a *p*-value denoting the significant difference and pair-wise comparisons.

Eosinophils from all study groups significantly stimulated ASMC and PF-induced collagen gel contraction compared to control cells, *p* < 0.001. The 24 h incubation with AA and SNEA eosinophils significantly promoted ASMC-induced collagen gel disk shrinkage compared to the HS eosinophil effect, respectively, 16.8 ± 1.1 vs. 10.7 ± 1.9 percentage of control ASMC, *p* < 0.05, and 33.9 ± 2.3 vs. 10.7 ± 1.9 percentage of control ASMC, *p* < 0.0001 (Figure 5). Furthermore, the SNEA eosinophils significantly increased ASMC contraction compared to the AA eosinophils effect, *p* < 0.0001. Regarding PF-induced collagen gel disk shrinkage, the AA and SNEA eosinophils had a significantly greater effect compared to HS group eosinophils, respectively, 19.3 ± 2.4 vs. 5.6 ± 1.4 percentage of control PF cells, *p* < 0.0001; and 21.4 ± 2.8 vs. 5.6 ± 1.4 percentage of control PF cells, *p* < 0.001 (Figure 5). However, the AA and SNEA eosinophils’ effect on PF contractility had no significant difference.

The subjects’ serum effect on ASMC and PF ability to contract collagen gel disk was examined. We found that serum of all study groups significantly promoted ASMC and PF to contract collagen gel disk compared to control cells, *p* < 0.01 (Figure 5A,B). ASMC-induced collagen gel shrinkage was significantly lower when incubated only with subjects’ serum compared to the eosinophil effect in AA and SNEA groups, respectively, 11.1 ± 1.4 vs. 16.8 ± 1.1 percentage of control ASMC, *p* < 0.05; and 24.3 ± 2.5 vs. 33.9 ± 2.3 percentage of control ASMC, *p* < 0.05. In addition, the combined eosinophil and the subjects’ serum effect was significantly higher compared with the effect of only serum in AA and SNEA groups, respectively, 23.3 ± 2.7 vs. 11.1 ± 1.4 percentage of control ASMC, *p* < 0.05; and 37.8 ± 3.1 vs. 24.3 ± 2.5 percentage of control ASMC, *p* < 0.05 (Figure 5A). However, significant differences between only eosinophil and combined eosinophil and the subjects’ serum effect on ASMC-induced collagen gel shrinkage were not found in all study groups.

The significant combined effect of eosinophils and subjects’ serum that significantly increased PF-induced collagen gel shrinkage compared with only eosinophil effect was found in AA and HS groups, respectively, 35.1 ± 3.8 vs. 19.3 ± 2.4 percentage of control PF cells, *p* < 0.05; and 14.3 ± 1.0 vs. 5.6 ± 1.4 percentage of control PF cells, *p* < 0.05. A significant difference between the eosinophil and serum combined effect and only serum was found in the AA and HS group, respectively, 35.1 ± 3.8 vs. 19.3 ± 6.8 percentage of control PF cells, *p* < 0.05; and 14.3 ± 1.0 vs. 5.7 ± 1.0 percentage of control PF cells, *p* < 0.05. SNEA serum and combined eosinophils and serum effect had a similar PF-induced collagen gel contraction promoting effect to that of eosinophils alone.

### 3.3. Migration of ASMC and PF after Incubation with Eosinophils

**Figure 6:**
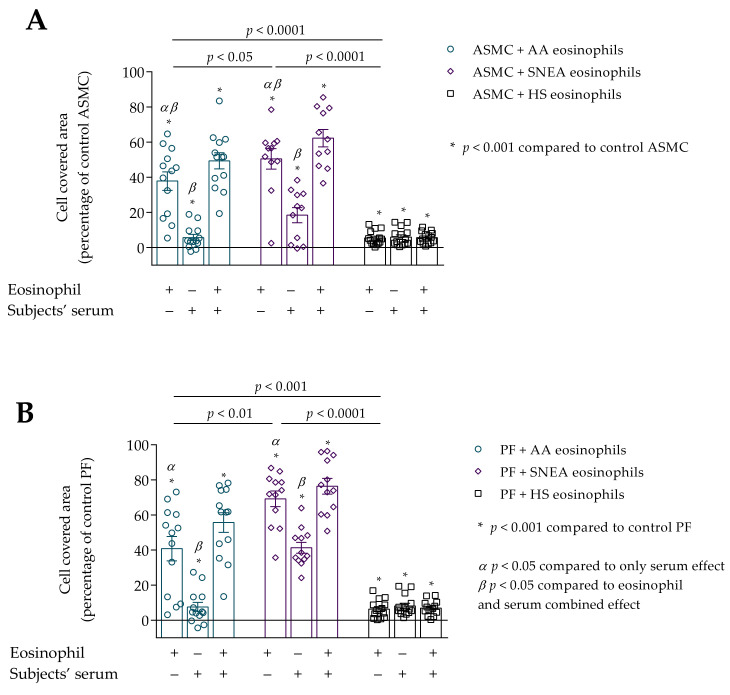
(**A**)–Migration of ASMC after incubation with eosinophils and/or subjects’ serum. (**B**)–Migration of PF after incubation with eosinophils and/or subjects’ serum. Data presented as cell covered area, as a percentage of control ASMC. Added blood serum of each investigated subject to individual combined cultures: 2% *v*/*v*. AA *n* = 13; SNEA *n* = 11; HS *n* = 14. AA—allergic asthma; ASMC—airway smooth muscle cells; HS—healthy subject; PF—pulmonary fibroblast; SNEA—severe non-allergic eosinophilic asthma. *α p* < 0.05 compared to the only serum effect; *β p* < 0.05 compared to the eosinophil and serum combined effect; A—* *p* < 0.001 compared to control ASMC, B—* *p* < 0.001 compared to control PF. Statistical analysis between investigated groups—Kruskal-Wallis test and post hoc Mann–Whitney two-sided *U*-test (independent data); within one study group—Friedman test for multiple comparison within the group and the post hoc Wilcoxon matched-pairs signed-rank two-sided test (dependent data). Significant differences were found in the eosinophil effect on ASMC and PF migration between investigated groups by Kruskal-Wallis test, respectively, χ^2^ = 22.33, df = 2, *p* < 0.0001 and χ^2^ = 25.93, df = 2, *p* < 0.0001. Significant differences of ASMC migration were found in AA and SNEA groups by Friedman test, respectively χ^2^ = 19.08, df = 2, *p* < 0.0001; χ^2^ = 16.55, df = 2, *p* < 0.0001. Significant differences of PF migration were found in AA and SNEA groups by Friedman test, respectively, χ^2^ = 19.08, df = 2, *p* < 0.0001; and χ^2^ = 14.60, df = 2, *p* = 0.0007. Lines connect comparison groups with a *p*-value denoting the significant difference in pair-wise comparisons.

Eosinophils from all study groups significantly promoted migration of ASMC and PF compared to control cells, *p* < 0.001. Significant differences were found in the eosinophil effect on ASMC and PF migration between investigated groups, respectively, *p* < 0.0001 and *p* < 0.0001. Migration of ASMC significantly increased after incubation with AA and SNEA eosinophils compared to HS eosinophils, respectively, 38.9 ± 5.3 vs. 5.4 ± 1.1 percentage of control ASMC, *p* < 0.0001; and 50.5 ± 5.8 vs. 5.4 ± 1.1 percentage of control ASMC, *p* < 0.0001 (Figure 6). Furthermore, the SNEA eosinophils significantly affected ASMC migration, compared to the AA eosinophil effect, *p* < 0.05. The same tendency was found in PF migration as that in AA, and SNEA eosinophils significantly increased PF migration compared to the HS eosinophil effect, respectively, 40.8 ± 6.9 vs. 6.3 ± 1.4 percentage of control PF cells, *p* < 0.001; and 69.2 ± 4.5 vs. 6.3 ± 1.4 percentage of control PF cells, *p* < 0.0001. The PF migration was significantly more intensive when incubated with SNEA eosinophils than AA eosinophils, *p* < 0.01.

We found that subjects’ serum of all study groups significantly increased ASMC and PF migration compared to control cells, *p* < 0.001 (Figure 6A,B). The serum effect on ASMC migration was significantly lower compared to the eosinophil effect in AA and SNEA groups, respectively, 37.9 ± 5.3 vs. 5.8 ± 1.8 percentage of control ASMC, *p* < 0.05; and 50.5 ± 5.8 vs. 18.5 ± 4.3 percentage of control ASMC, *p* < 0.05. In addition, the combined eosinophil and serum effect was significantly higher in AA and SNEA groups compared to only the eosinophils effect, respectively, 49.4 ± 4.6 vs. 38.9 ± 5.3 percentage of control ASMC, *p* < 0.05; 62.3 ± 5.0 vs. 50.5 ± 5.8 percentage from control ASMC, *p* < 0.05; and compared to the only serum effect, respectively, 49.4 ± 4.6 vs. 5.8 ± 1.8 percentage of control ASMC, *p* < 0.05; 62.3 ± 5.0 vs. 18.5 ± 4.3 percentage of control ASMC, *p* < 0.05.

The migration of PF cells was significantly higher when incubated with AA and SNEA eosinophils compared to the serum effect, respectively, 40.8 ± 6.9 vs. 7.6 ± 2.7 percentage of control PF, *p* < 0.05; and 69.2 ± 4.5 vs. 41.3 ± 3.1 percentage of control PF cells, *p* < 0.05. The migration was more intensive after incubation with eosinophils and serum in AA and SNEA groups compared to the only serum effect, respectively, 55.7 ± 5.6 vs. 7.6 ± 2.7 percentage of control PF cells, *p* < 0.05; and 76.4 ± 4.5 vs. 41.3 ± 3.1 percentage of control PF cells, *p* < 0.05. The addition of AA serum to medium with subjects’ eosinophils significantly increased the PF migration compared to only the eosinophil effect, 40.8 ± 6.9 vs. 55.7 ± 5.6 percentage of control PF cells, *p* < 0.05.

### 3.4. Gene Expression in ASMC and PF

**Figure 7:**
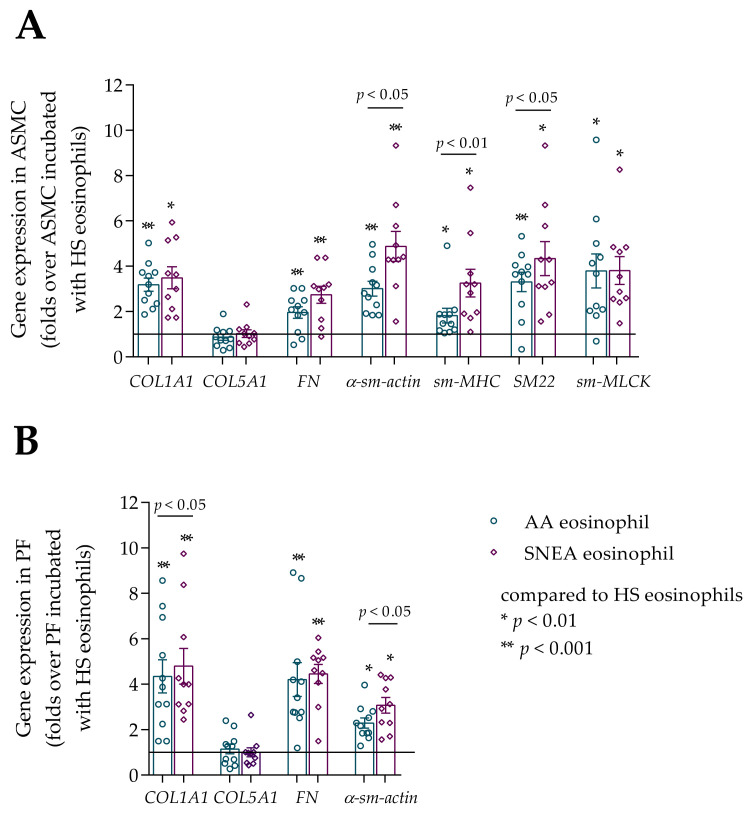
(**A**)–Gene expression in ASMC after incubation with eosinophils; (**B**)–Gene expression in PF after incubation with eosinophils. Data presented as folds over the HS eosinophil effect as mean ± SEM. AA—allergic asthma patient; SNEA—severe non-allergic eosinophilic asthma patient; ASMC—airway smooth muscle cells; PF–pulmonary fibroblast; HS—healthy subject; *COL1A1*—collagen I α 1; *COL5A1*—collagen V α 1; *FN*—fibronectin; α-sm-actin—α smooth muscle actin; *sm*-*MHC*—smooth muscle myosin heavy chain; *SM22*—transgelin; *sm-MLCK*—smooth muscle myosin light chain kinase. * *p* < 0.01 compared to HS eosinophils; ** *p* < 0.001 compared to HS eosinophils. AA *n* = 11; SNEA *n* = 10; HS *n* = 8. Statistical analysis between investigated groups—Mann–Whitney two-sided U-test (independent data); Wilcoxon signed-rank test was used for gene expression analysis against ASMC or PF control. Lines connect comparison groups with a *p*-value denoting the significant difference in pair-wise comparisons.

Gene expression of contractile markers (α-sm-actin, *sm*-*MHC*, *SM22*, *sm-MLCK* for ASMC, and α-sm-actin for PF) and main fibril ECM proteins (*COL1A1* and *FN*) in ASMC and PF was significantly higher when incubated with AA and SNEA eosinophils compared with the HS eosinophil effect, *p* < 0.05. However, eosinophils did not affect *COL5A1* gene expression in ASMC and PF in any study group. The α-sm-actin, *sm*-*MHC*, *SM22* gene expression was significantly higher in ASMC incubated with SNEA eosinophils compared to the AA eosinophil effect; accordingly, α-sm-actin 4.9 ± 0.7 vs. 3.0 ± 0.3 fold over ASMC incubated with HS eosinophils, *p* < 0.05; *sm*-*MHC* 3.3 ± 0.6 vs. 1.8 ± 0.3 fold over ASMC incubated with HS eosinophils; *SM22* 4.3 ± 0.8 vs. 3.3 ± 0.4 fold over ASMC incubated with HS eosinophils, *p* < 0.05; whereas a significant difference between the AA and SNEA eosinophil effect on *COL1A1*, *FN*, *sm-MLCK* was not found (Figure 7A). Gene expression of *COL1A1* and α-sm-actin in PF was significantly higher after incubation with SNEA eosinophils compared to the AA eosinophil effect, respectively, *COL1A1* 4.8 ± 0.8 vs. 4.3 ± 0.7 fold over PF incubated with HS eosinophils, *p* < 0.05; α-sm-actin 3.1 ± 0.3 vs. 2.3 ± 0.2 fold over PF incubated with HS eosinophils, *p* < 0.05; whereas significant differences between AA and SNEA eosinophil effects on *FN* expression in PF were not found (Figure 7B).

### 3.5. The Effect of D. pteronyssinus Allergen Activated Eosinophils In Vivo on the Contractility of ASMC and PF

**Figure 8:**
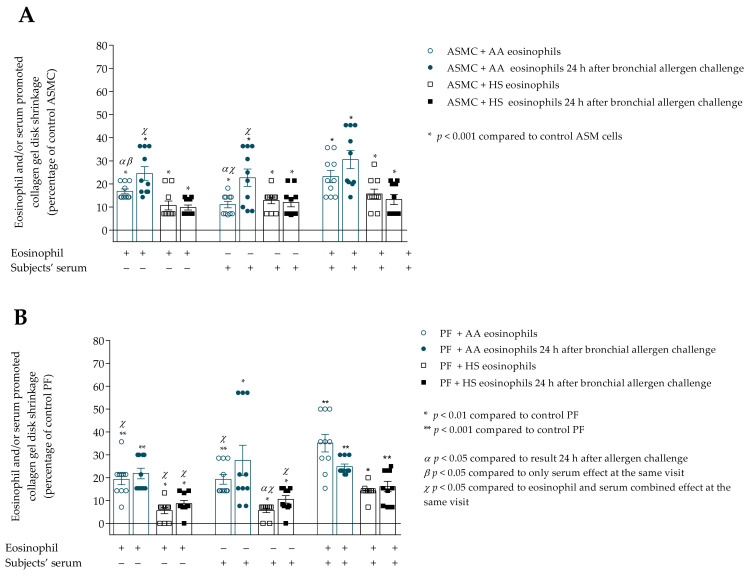
(**A**)–Contraction of collagen gel disk with ASMC after incubation with eosinophils and/or subjects’ serum 24 h after bronchial allergen challenge with D. pteronyssinus; (**B**)–Contraction of collagen gel disk with PF after incubation with eosinophils and/or subjects’ serum 24 h after bronchial allergen challenge with *D. pteronyssinus* allergen. Data presented as a percentage of control ASMC that were not incubated with eosinophils. Added blood serum of each investigated subject to individual combined cultures: 2% *v*/*v*. ASMC—airway smooth muscle cells; HS—healthy subject; AA—allergic asthma; PF—pulmonary fibroblast. α—*p* < 0.05 compared to results 24h after allergen challenge; β—*p* < 0.05 compared to the only serum effect at the same visit; χ—*p* < 0.05 compared to the eosinophil and serum combined effect at the same visit; A—* *p* < 0.001 compared to control ASMC; B—* *p* < 0.01 compared to control PF; ** *p* < 0.001 compared to control PF. AA *n* = 10; HS *n* = 10. Statistical analysis within one study group—Friedman test for multiple comparison within the group and the post hoc Wilcoxon matched-pairs signed-rank two-sided test (dependent data). Significant differences of ASMC-induced collagen gel shrinkage were found in AA group 24 h after bronchial allergen challenge by Friedman test, χ^2^ = 12.96, df = 2, *p* = 0.0005. Significant differences of PF-induced collagen gel shrinkage were found in HS group 24 h after bronchial allergen challenge by Friedman test, χ^2^ = 9.929, df = 2, *p* = 0.0038. The results from Figure 5A,B of AA and HS patients were re-used as the baseline (before allergen challenge) result.

*D. pteronyssinus* allergen-activated AA eosinophils in vivo significantly increased ASMC-induced collagen gel disk shrinkage compared to baseline visit results (before allergen challenge), 24.6 ± 2.9 vs. 16.8 ± 1.1 percentage of control ASMC, *p* < 0.05 (Figure 8A). The same tendency was found in the serum effect: 24 h after allergen challenge, the serum effect on ASMC-induced collagen gel contraction was significantly increased compared to baseline visit results, 22.7 ± 3.8 vs. 11.1 ± 1.4 percentage of control ASMC, *p* < 0.05. 

The combined eosinophil and serum effect was found in the AA group 24 h after bronchial allergen challenge, and significantly increased ASMC-induced collagen gel disk shrinkage compared to only eosinophil, and in addition to the only serum effect, respectively, 30.2 ± 3.9 vs. 24.6 ± 2.9 percentage of control ASMC, *p* < 0.05; and 30.6 ± 3.9 vs. 22.7 ± 3.8 percentage of control ASMC, *p* < 0.05.

Regarding the allergen-activated eosinophil effect on PF-induced collagen gel contraction, we found no significant differences in the AA group (Figure 8B). PF-induced collagen gel shrinkage was significantly increased after incubation with HS group serum 24 h after allergen challenge compared to baseline results, 10.6 ± 1.6 vs. 5.7 ± 1.0 percentage of control PF cells, *p* < 0.05. Furthermore, the HS combined eosinophil and serum effect on PF contractility was significantly higher compared with only eosinophils, in addition to the only serum effect 24 h after bronchial challenge with the *D. pteronyssinus* allergen, respectively, 16.1 ± 2.3 vs. 8.7 ± 1.4 percentage of control PF cells, *p* < 0.05; and 16.1 ± 2.3 vs. 10.6 ± 1.6 percentage of control PF cells, *p* < 0.05.

### 3.6. The Effect of D. pteronyssinus Allergen Activated Eosinophils In Vivo to the Migration of ASMC and PF

**Figure 9:**
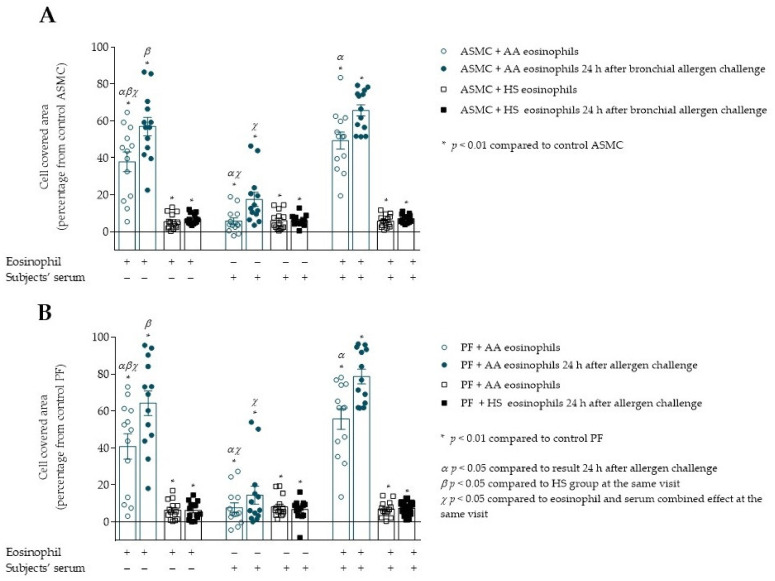
(**A**)–Migration of ASMC after incubation with allergen-activated eosinophils and/or subjects’ serum. (**B**)–Migration of PF after incubation with allergen-activated eosinophils and/or subjects’ serum. Data presented as cell covered area, as a percentage of control cells. Added blood serum of each investigated subject to individual combined cultures: 2% *v*/*v*. AA *n* = 13; HS *n* = 14. AA—allergic asthma; ASMC—airway smooth muscle cells; HS—healthy subject; PF—pulmonary fibroblast. α—*p* < 0.05 compared to results 24h after allergen challenge; *β*—*p* < 0.05 compared to the only serum effect at the same visit; χ—*p* < 0.05 compared to the eosinophil and serum combined effect at the same visit; A—* *p* < 0.001 compared to control ASMC; B—* *p* < 0.01 compared to control PF cells. Statistical analysis within one study group—Friedman test for multiple comparison within the group and the post hoc Wilcoxon matched-pairs signed-rank two-sided test (dependent data). Significant differences of ASMC and PF migration were found in AA group 24 h after bronchial allergen challenge by Friedman test, respectively, χ^2^ = 17.23, df = 2, *p* = 0.0002; χ^2^ = 18.00, df = 2, *p* = 0.0001. The results from Figure 6A,B of AA and HS patients were re-used as the baseline (before allergen challenge) result.

Migration of ASMC was significantly increased after incubation with allergen-activated asthmatic eosinophils compared to the baseline eosinophil effect, 56.9 ± 5.0 vs. 37.9 ± 5.3 percentage of control ASMC, *p* < 0.05 (Figure 9A). The AA serum had a significantly greater effect after bronchial allergen challenge on ASM migration compared to the baseline, 17.6 ± 3.8 vs. 5.8 ± 1.8 percentage of control ASMC, *p* < 0.05. Asthmatic eosinophil effect was significantly higher to ASMC migration compared to serum effect, 56.9 ± 5.0 vs. 17.6 ± 3.8 percentage of control ASMC, *p* < 0.05; and combined eosinophil and serum effect was significantly increased compared to the only serum effect on ASMC migration, 65.7 ± 3.0 vs. 17.6 ± 3.8 percentage of control ASMC, *p* < 0.05.

PF migration was significantly increased when incubated with allergen-activated asthmatic eosinophils compared to the baseline result, 64.2 ± 6.7 vs. 40.8 ± 6.9 percentage of control PF cells, *p* < 0.05 (Figure 9B). In addition, the serum from AA patients 24 h after bronchial allergen challenge had a more significant effect on PF migration than at baseline, 14.5 ± 4.9 vs. 7.6 ± 2.7 percentage of control PF cells, *p* < 0.05. Furthermore, 24 h after the bronchial allergen challenge, combined AA eosinophils and patients’ serum significantly increased PF migration compared to the baseline combined eosinophil and serum effect, 78.7 ± 4.0 vs. 55.7 ± 5.6 percentage of control PF cells, *p* < 0.05. Eosinophil effect on PF migration was significantly increased compared to serum effect, 64.2 ± 6.7 vs. 14.45 ± 4.9 percentage of control PF, *p* < 0.05; and combined eosinophil and serum effect was significantly higher compared to only serum effect, 78.7 ± 4.0 vs. 14.5 ± 4.9 percentage of control PF, *p* < 0.05.

### 3.7. The Effect of Allergen-Activated Eosinophils on Gene Expression in ASMC and PF

**Figure 10:**
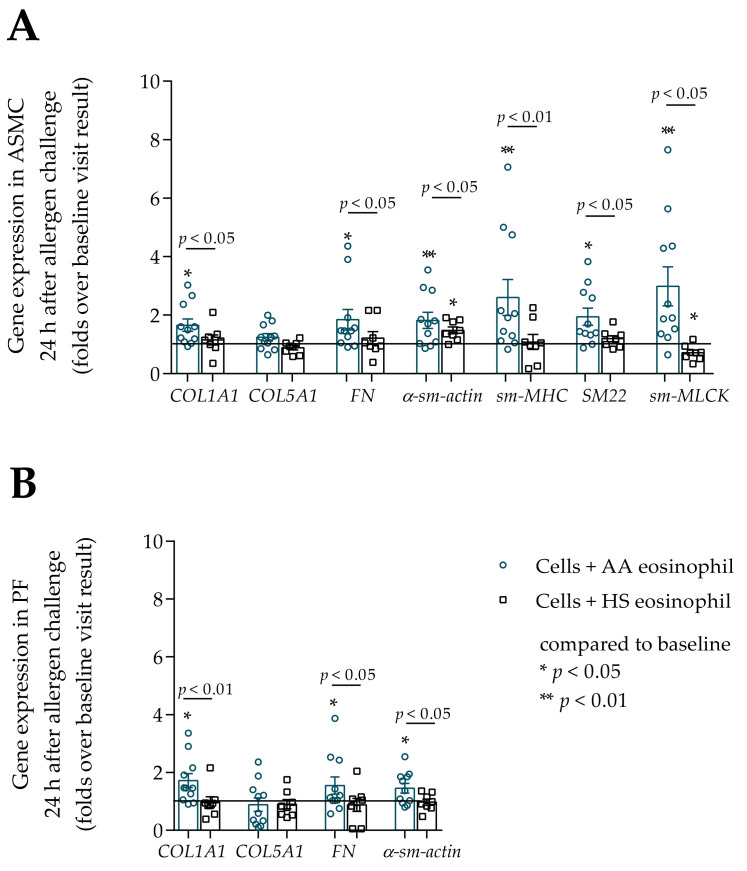
(**A**)–The change in gene expression in ASMC 24 h after bronchial allergen challenge with *D. pteronyssinus*, (**B**)–The change in gene expression in PF cells 24 h after bronchial allergen challenge with *D. pteronyssinus*. Data presented as folds over baseline visit result as mean ± SEM. Added blood serum of each investigated subject to individual combined cultures: 2% *v*/*v*. AA—allergic asthma patient; ASMC—airway smooth muscle cells; PF—pulmonary fibroblast; HS—healthy subject; *COL1A1*—collagen I α 1; *COL5A1*—collagen V α 1; *FN*—fibronectin; α-sm-actin—α smooth muscle actin; *sm*-*MHC*—smooth muscle myosin heavy chain; *SM22*—transgelin; *sm-MLCK*—smooth muscle myosin light chain kinase. AA *n* = 11; HS *n* = 8. * *p* < 0.05 compared to control cells; ** *p* < 0.01. Statistical analysis between investigated groups—Mann–Whitney two-sided U-test (independent data); Wilcoxon signed-rank test was used for gene expression analysis against ASMC or PF control. Lines connect comparison groups with a *p*-value denoting the significant difference in pair-wise comparisons.

Twenty-four hours after bronchial allergen challenge, the gene expression of all previously evaluated genes significantly increased in ASMC and PF cells compared to baseline results in the AA group, *p* < 0.05. However, allergen-activated eosinophils did not affect *COL5A1* gene expression in ASMC and PF cells. The gene expression of *COL1A1*, *FN*, *α*-*sm*-*actin*, *sm*-*MHC*, *SM22*, and *sm*-*MLCK* in ASMC after incubation with allergen-activated AA eosinophils was significantly higher compared to the HS eosinophil effect, respectively, *COL1A1* 1.7 ± 0.2 vs. 1.2 ± 0.2 fold over baseline, *p* < 0.05; *FN* 1.9 ± 0.4 vs. 1.2 ± 0.2 fold over baseline, *p* < 0.05; α-sm-actin 1.8 ± 0.3 vs. 1.5 ± 0.1 fold over baseline, *p* < 0.05; *sm*-*MHC* 2.6 ± 0.6 vs. 1.1 ± 0.3 fold over baseline, *p* < 0.01; *SM22* 2.0 ± 0.3 vs. 1.2 ± 0.1 fold over baseline, *p* < 0.05; *sm-MLCK* 3.0 ± 0.7 vs. 0.7 ± 0.1 fold over baseline, *p* < 0.05 (Figure 10A). Gene expression of *COL1A1*, *FN*, and α-sm-actin in PF cells was significantly increased after incubation with allergen-activated AA eosinophils compared to the HS eosinophil effect, respectively, *COL1A1* 1.7 ± 0.2 vs. 1.0 ± 0.2 fold over baseline, *p* < 0.05; *FN* 1.6 ± 0.3 vs. 0.9 ± 0.2 fold over baseline, *p* < 0.05; α-sm-actin 1.5 ± 0.2 vs. 1.0 ± 0.1 fold over baseline, *p* < 0.05 (Figure 10B).

## 4. Discussion

The study results showed that asthmatic eosinophils promoted ASMC and PF-induced collagen gel shrinkage, increased migration of ASMC and PF cells, and promoted their differentiation into a more contractile phenotype. Additionally, SNEA eosinophils had a higher impact on ASMCs’ ability to contract collagen gel and both cell lines’ migration than AA eosinophils. The gene expression of main ECM proteins such as *COL1A1*, *FN,* and contractile phenotype markers such as α-sm-actin, sm-MHC, SM22, and sm-MLCK for ASMC, and α-sm-actin for PF cells, increased in both cell lines after co-culture with asthmatic eosinophils; the highest effect was produced with SNEA eosinophils. Furthermore, in vivo allergen-activated AA eosinophils further stimulated the contractility of ASMC, whereas migration and gene expression stimulated that of both cell lines.

Type 2 asthma is characterized as a chronic eosinophilic airway inflammatory disease with blood and airway eosinophilia [20,21]. Previously we showed that asthmatic eosinophils’ adhesion to structural lung cells was increased, which suggests that eosinophils may adhere to ASMC and PF during their migration from blood to airway lumen [16]. Adhered eosinophils degranulate and secrete various growth factors causing behavior changes of ASMC, PF, and epithelial cells that result in airway remodeling [22]. Airway remodeling is broadly characterized as the reorganization of structural cell composition, impaired cellular function, and ECM protein production dysregulation. Increased contractility and migration of ASMC and PF are part of airway inflammatory processes that contribute to airway remodeling in asthma [23]; however, little is known about eosinophils’ role in these processes.

The prime function of ASMC is to regulate airway tone via a balance between the contraction and relaxation in response to local or circulating factors [24]. ASMC are recognized as regulatory cells due to the production of ECM proteins, growth factors, and pro- and anti-inflammatory mediators, thus influencing other airway structural cells’ proliferation, migration, and apoptosis [24]. Several studies focused on the nature of asthmatic ASMC showed that greater force, contraction, and extension were generated than in healthy ASMC [5,6,25]. Furthermore, asthmatic eosinophils’ on ASMC-induced collagen gel shrinkage was greater than that of healthy eosinophils, and the effect of SNEA eosinophils was greater than that of AA eosinophils. These results may be explained by eosinophil activity differences in different asthma phenotypes. It was found that SNEA eosinophils increased gene expression of integrins and *β*-chain signaling cytokine receptors compared to AA eosinophils, showing that SNEA eosinophils are more sensitive to stimulants while also being more active and able to produce more proinflammatory mediators [26,27,28].

ASMC, as in the case of other smooth muscle cells, contains actin and myosin filaments arranged as opposing bundles [29]. Contraction is initiated by a Ca2+-calmodulin interaction that stimulates phosphorylation of the myosin light chain, and this process depends on RhoA/Rho signaling pathway activity. However, abnormal ASMC contraction is found in such diseases as asthma, where smooth muscle cells’ contraction is augmented or spastic, resulting in bronchoconstriction [30]. This may be explained by increased eosinophilic inflammation in airways because eosinophils and activated ASMC produce high levels of TGF-*β* that are closely related to RhoA/Rho signaling pathway activity [31].

The contractility of fibroblasts is associated with differentiation to a more productive and contractile phenotype known as myofibroblasts. Myofibroblasts are mesenchymal cells that are often described as a cross between fibroblasts and smooth muscle cells because they have increased α-sm-actin expression, which is visible in cells as stress fibers [32]. The apparatus of myofibroblasts contractility is composed of actin-rich bundles of microfilaments that are terminated with focal adhesions [32]. Actin stress fibers activated by soluble factors communicate with ECM proteins via focal adhesions that result in cell contraction [33]. PF-induced collagen gel contraction is promoted by adding eosinophil to the medium, especially asthmatic. Asthmatic eosinophils are a stress factor for fibroblasts, resulting in PF differentiation into myofibroblasts and contraction.

The *FN* expression may explain why the SNEA and AA eosinophil effect on PF-promoted collagen gel shrinkage did not differ in our study; we found that SNEA and AA eosinophils’ effect on *FN* gene expression in PF was similar. Together with α-sm-actin and integrins, the intracellular fibronectin is crucial for myofibroblasts’ contraction [34]. Furthermore, contractility may be regulated by high doses of inhaled steroids that inhibit blood eosinophil activation and adhesion, thus potentially weakening the SNEA eosinophil effect on PF contractility [35,36,37,38]. Zhang et al. used a dynamic microscale platform to evaluate fibroblast contractility using collagen I and found that eosinophils induce PF contractility [39]. The human myeloid leukemic eosinophil cell line used in this study experiment cannot reflect the effect of asthmatic blood eosinophils on structural lung cells.

Airway narrowing is associated with ASMC’s ability to contract due to easier binding of actin and myosin, their hypertrophy, hyperplasia, excessive ECM protein deposition, and edema [40,41,42,43,44,45,46]. ASMC bundle thickening, responsible for the reduced airway caliber and possibly associated with exaggerated contractility, may represent a relevant factor contributing to airway hyperresponsiveness [47]. It was previously shown that the ASMC layer’s increased mass is indeed correlated with airway responsiveness to methacholine and asthma severity [48,49]. Contractility and migration are closely related processes. Cell migration results from the cascade of reactions in the activation of contractile apparatus proteins and is a highly explicit molecular machinery that coordinates protrusion and contraction [9]. It is suggested that ASMC migration contributes to their hyperplasia, increasing mass in airways [9]. Activated ASMC produces various cytokines such as interleukin (IL)-4, IL-5, IL-13, and thymic stromal lymphopoietin (TSLP); and chemokines such as CCL5, CCL11, CXCL8, and CXCL10, which cause autocrine ASMC activation resulting in enhanced proliferation and migration [9]. We found that asthmatic eosinophils promoted ASMC migration, and SNEA eosinophils had a greater influence on migration than AA eosinophils in vitro.

The interaction between ECM proteins and cells supports physiological cells’ activities and plays an important role in pathological processes such as wound healing, tissue fibrosis, and scar formation [50]. In asthma, it is also associated with augmented migratory properties of PF [51]. Eosinophils secrete pro-inflammatory mediators, and studies suggest that increased eosinophil cationic protein and TGF-*β* can stimulate PF migration, resulting in airway basement membrane thickening [52,53]. Asthmatic eosinophils, particularly SNEA eosinophils, promote PF migration, which may be explained by more activated eosinophils and increased secretion. Although contractility and migration are closely related processes, chemoattractants that are abundantly secreted by eosinophils and wounded PF are crucial for PF migration. Furthermore, the biopsies revealed different eosinophil activity in airways, with eosinophils of patients with AA, allergic rhinitis, or nasal polyposis having higher degranulation intensity than in other airway diseases [54]. As mentioned previously, eosinophils can adhere to structural lung cells such as PF, resulting in eosinophil secretion of pro-inflammatory and fibroblast migration-promoting mediators [16,55,56].ASMC can be divided into contractile, proliferative, and synthetic phenotypes that show the domination of different cell properties [25]. It has been shown that ASMC increases proliferation and ECM production simultaneously, thus suggesting that proliferative and synthetic phenotypes overlap [57]. The contractile ASMC phenotype is characterized by increased expression of contractile apparatus proteins. Furthermore, the triggers such as inflammatory mediators and ECM proteins responsible for ASMC phenotype switching are the subject of discussion; however, it is agreed that this process contributes to asthma pathogenesis [25,58,59]. Whether modulation from contractile to proliferative–synthetic also occurs in vivo in humans remains to be established [60,61]. It was shown that freshly isolated ASMC are contractile, and under serum-rich conditions, they become proliferative–synthetic [59]. Furthermore, it was demonstrated that asthmatic ASMC differ from non-asthmatic ASMC in their behavior: asthmatic ASMC may be simultaneously hypercontractile, hypersynthetic, and hyperproliferative [2,3,4,62].

The contractile ASMC phenotype has increased expression of *MLCK*, *MHC*, transgelin (*SM22*), and *α-sm-actin*, whereas enhanced proliferative potential and the expression of *COL1A1*, *FN*, connective tissue growth factor (*CTGF*), *CXC10*, and *CCL11* characterizes proliferative–synthetic ASMC [25]. We found that the gene expression of *α-sm-actin*, *sm*-*MHC*, *SM22*, *sm-MLCK, COL1A1*, and *FN* increased in ASMC after incubation with asthmatic eosinophils, showing that eosinophils promote ASMC differentiation, which demonstrates the presence of both contractile and proliferative–synthetic ASMC phenotypes. The idea of phenotype switching implies that contractile and proliferative capacities are the opposite to each other [57]. Perhaps these phenotypes of ASMC coexist in airways, forming a heterogeneous population of ASMC. However, the ratio at which these phenotypes exist in vivo and in vitro is unknown [25]. This is important for future investigations, which should aim to understand asthma pathogenesis better.

PF resides in highly complex multicellular environments, usually near the epithelium and endothelium. Fibroblasts are considered the primary source of ECM proteins that provide a scaffold for cells and play critical roles in determining the phenotype and function of structural lung cells in health and disease via quantitative and qualitative molecular composition stiffness [63]. In asthma, PF contributes to injury responses, causing the loss of normal lung tissue architecture and function, thereby leading to fibrosis and impaired airway tissue homeostasis. The most important factor for fibroblasts’ differentiation into myofibroblasts is TGF-*β*, which is highly expressed in asthmatic airways [64,65]. The main marker of fibroblasts’ differentiation into myofibroblasts is *α*-*sm*-*actin*, *COL1A1*, and *FN* expression under the influence of TGF-*β* when the stress fibers and focal adhesion complexes form [66,67,68]. The study showed that asthmatic eosinophils are responsible for increased gene expression of *α*-*sm*-*actin*, *COL1A1*, and *FN* in PF, which characterizes their differentiation into myofibroblasts. Furthermore, these ECM proteins are necessary for the migration of myofibroblasts [69,70,71]. Collagen V is a fibrillar collagen that forms fibrils and supports ECM organization [72]. We suggest that eosinophils are not responsible for *COL5A1* expression because we did not find that eosinophils change *COL5A1* expression in ASMC and PF. Thus, the increased PF contractility and migration may be associated with differentiation into myofibroblasts and eosinophil-promoted *COL1A1* and *FN* expression in PF.

The serum is a source of a wide variety of growth factors, interleukins, and other biologically active mediators; however, in diseases such as asthma, increased levels of pro-inflammatory mediators are found [73,74,75]. The experiments showed that asthmatic serum increased contractility and migration of ASMC and PF compared to control cells. The addition of AA patients’ serum to AA eosinophils and ASMC or PF combined cultures promoted PF contractility and migration of both cell lines. Furthermore, the migration of ASMC was higher after adding SNEA serum to the combined culture with SNEA eosinophils; however, there was no significant effect on ASMC and PF contractility and PF migration, and it may be presumed that this was due to the influence of the high doses of inhaled steroids that were used by SNEA patients [76,77,78]. Furthermore, this result shows that AA, SNEA, and HS serum had different quantitative compositions of contractility and migration-stimulating factors. Increased IL-4, IL-5, IL-13, IL-33, TSLP, TGF-*β*, eosinophil granule-derived protein, and platelet-derived growth factor (PDGF) levels promote migration of structural lung cells via actin polymerization, cell polarization, and inflammation in the airways [79,80,81]. Furthermore, TGF-*β* also is responsible for increased cell motility [82]. Our previous study showed that AA eosinophils caused an increase in TGF-*β*1 gene expression in ASMC and higher TGF-*β*1 concentration in the medium [83]. Furthermore, it was shown that ASMC behavior depends on different conditions; for example, in one study, insulin increased the expression of contractile phenotype markers and ECM molecules such as collagen I via the Rho signaling pathway activation [84]. As another study showed, asthma patients have poor glycemic control caused by hyperinsulinemia, promoting ASMC differentiation into the contractile phenotype [85]. Furthermore, serum addition shows that eosinophils may be activated and have a compound effect on ASMC and PF behavior.

Asthma is a heterogeneous disease that is usually triggered by environmental factors such as allergens. The allergen challenge with *D. pteronyssinus* for AA patients was used to mimic acute asthma attacks. Allergen-activated eosinophils augmented the contractility of ASMC and the migration of ASMC and PF, and increased gene expression of differentiation markers and ECM proteins in both cell lines compared to the baseline result. This may be explained by the activation of eosinophils and increased secretion of mediators. Allergen-activated eosinophils and epithelial cells, in addition to ASMC and PF themselves, produce more ASMC contractility and ASMC and PF migration-promoting mediators [86,87]. Allergen challenge studies with animal AA models and AA patients suggest increased differentiation into contractile ASMC and PF phenotypes, leading to increased contractility and migration [88,89]. Our study shows that the allergen-activated eosinophils are more active and capable of promoting gene expression, differentiation into the contractile phenotype, and migration, thus participating in asthma pathogenesis.

A possible drawback of our study could be that we were unable to evaluate the single-cell contraction potency. However, we aimed to evaluate the asthmatic and healthy eosinophil effect on ASMC and PF contractility and differentiation into the contractile phenotype, and collagen gel assay allows to evaluate the influence of eosinophils on ASMC/PF-induced collagen gel shrinkage [18]. Several studies confirmed that collagen gel assay is an appropriate method to evaluate the ASMC and PF contractility in changing environmental conditions such as adding contraction stimulating or inhibiting factors, thus providing a model for tissue contraction [90,91,92,93]. In the current study, we used eosinophils as a contraction of structural lung cells stimulating factor. Furthermore, the serum-free conditions were used for collagen gel and wound healing assay experiments, thus minimizing the influence of ASMC and PF proliferation as serum starvation inhibits the proliferation of these cells.

In conclusion, asthmatic eosinophils change ASMC and PF activity by increasing their contractility and migration, thus contributing to airway remodeling. Our data could provide a better understanding of eosinophils’ role in asthma pathogenesis and the effect of eosinophil-related changes on the behavior of lung structural cells, thus helping to provide more individualized treatment of asthma.

## Figures and Tables

**Table 1:** Inclusion and exclusion criteria for the study population.

	AA Patients (*n* = 13)	SNEA Patients (*n* = 11)	HS (*n* = 14)
Screening visit (for all groups)	- History and physical examination - Complete blood count - Spirometry - Methacholine challenge test - Skin prick test		
Inclusion criteria	- Asthma symptoms ≥ 1 year - A non-severe course of the disease - Positive skin prick test to *D. pteronyssinus* - Positive methacholine challenge test	- Asthma history ≥ 1 year - Negative skin prick test - Peripheral blood eosinophil count ≥0.3 × 10^9^/L - High doses of inhaled steroids and long-acting *β* agonists	- No chronic respiratory and other diseases - Negative skin prick test - Negative methacholine challenge test
Exclusion criteria (for all groups)	- Clinically significant allergy symptoms - Active airway infection ≤ 1 month prior to study - Asthma exacerbation ≤ 1 month prior to study - Use of oral steroids ≤1 month prior to study - Smoking		

AA—allergic asthma; HS—healthy subjects; SNEA—severe non-allergic eosinophilic asthma; *D. pteronyssinus*—*Dermatophagoides pteronyssinus.*

**Table 2:** Primers used for gene expression analysis.

Gene	Forward 5′–3′	Reverse 5′–3′
*18S*	CGCCGCTAGAGGTGAAATTC	TTGGCAAATGCTTTCGCTC
*α*-*sm*-*actin*	TGGGTGACGAAGCAC AGAGC	CTTCAGGGGCAACACGAAGC
*sm*-*MHC*	CGCCAAGAGACTCGTCTGG	TCTTTCCCAACCGTGACCTTC
*SM22*	AGGAGCGGCTGGTGGAGTGGAT	CATGTCAGTCTTGATGACCCCATAGT
*sm*-*MLCK*	GACTGCAAGATTGAAGGATAC	GTTTCCACAATGAGCTCTGC
*COL1A1*	TCGAGGAGGAAATTCCAATG	ACACACGTGCACCTCATCAT
*COL5A1*	GGCTCCCGAGAGCAACCT	CGGGACACTCACGAACGAA
*FN*	AGCCAGCAGATCGAGAACAT	TCTTGTCCTTGGGGTTCTTG

*18S*—reference gene; *α*-*sm*-*actin*—α smooth muscle actin gene; *sm*-*MHC*—smooth muscle myosin heavy chain gene; *SM22*—transgelin gene; *sm*-*MLCK*—smooth muscle myosin light chain kinase gene; *COL1A1*—collagen I α1 gene; *COL5A1*—collagen V α1 gene; *FN*—fibronectin gene.

**Table 3:** Demographic and clinical characteristics of the study population.

	AA Patients, *n* = 13	SNEA Patients, *n* = 11	HS, *n* = 14
Age, median (range), years	25.8 (18.0–40.0)	54.0 (28.0–80.0) * #	31.5 (23.0–59.0)
Sex, (male/female), *n*	7/5	6/5	4/10
BMI, kg/m^2^, median (range)	23.8 (17.3–40.1)	27.5 (17.5–37.3)	23.9 (17.0–34.4)
Sensitization to *D. pteronyssinus*/*D. farinae*/birch/5 grass mixture allergen, *n*	13/9/4/3	NR	NR
Wheel diameter by *D. pteronyssinus*, median (range), mm	8.4 (4.0–15.0)	0	0
PD_20M_, geometric mean (range), mg	0.16 (0.05–0.41)	ND	NR
FEV_1_, % of predicted	85.2 ± 11.5	51.3 ± 26.4 * #	107.0 ± 11.6
FEV_1_, L	3.6 ± 0.8	1.7 ± 1.3 * #	3.8 ± 0.6
Blood eosinophil count, × 10^9^/L	0.40 ± 0.24 *	0.63 ± 0.55 * #	0.19 ± 0.09
Blood eosinophil count, %	6.17 ± 3.97 *	10.30 ± 8.55 * #	2.92 ± 1.10
IgE, IU/mL	583.0 (88.9–4617.0) *	196.0 (11.2–795.0) * #	26.2 (3.0–67.4)

Data presented as a median (range), mean ± SD. AA—allergic asthma; F—female; FEV_1_—forced expiratory volume in 1 s; HS—healthy subject; IgE—immunoglobulin E; M—male; NR—not responded; ND—not done; PD_20M_—the provocation dose of methacholine causing a 20% decrease in FEV_1_; SNEA—severe non-allergic eosinophilic asthma. * *p* < 0.05 compared with HS group; # *p* < 0.05 compared with AA group. Statistical analysis between investigated groups Mann–Whitney two-sided *U*-test (independent data).

**Table 4:** Clinical characteristics of the study population 24 h after bronchial challenge with *D. pteronyssinus*.

	AA Patients, *n* = 13		HS, *n* = 14	
	Before the allergen challenge	24 h after allergen challenge	Before the allergen challenge	24 h after allergen challenge
Blood eosinophil count, × 10^9^/L	0.40 ± 0.24	0.47 ± 0.21 *	0.19 ± 0.09	0.14 ± 0.05
Blood eosinophil count, %	6.17 ± 3.97	7.08 ± 3.77 *	2.92 ± 1.10	3.07 ± 1.81
IgE, IU/mL	583 (88.9–4617.0)	837 (95.1–4325) *	26.2 (3.0–67.4)	27.6 (3.0–71.2)

Data presented as a median (range) or mean ± SD. AA—allergic asthma; HS—healthy subjects; IgE—immunoglobulin E. * *p* < 0.05 compared with baseline result at the same group. Statistical analysis—Mann–Whitney two-sided *U*-test (independent data); Wilcoxon matched-pairs signed-rank test (dependent data).

## Data Availability

All the data presented in this study are included in this article.

## Supplementary Materials

The following are available online at https://www.mdpi.com/article/10.3390/cells10061389/s1, Figure S1: Images of ASMC and PF at 0, 24, 48, and 72 h time points.

## Abbreviations

AA	Allergic asthma
ASMC	Airway smooth muscle cells
BMI	Body mass index
*COL1A1*	Collagen I alpha 1 gene
*COL5A1*	Collagen V alpha 1 gene
CTGF	Connective tissue growth factor
*D. pteronyssinus*	*Dermatophagoides pteronyssinus*
ECM	Extracellular matrix
FEV1	Forced expiratory volume in 1 s
*FN*	Fibronectin gene
HS	Healthy subject
hTERT	Human telomerase reverse transcriptase
IgE	Immunoglobin E
IL	Interleukin
PD_20M_	Provocative dose of methacholine causing a 20% drop in FEV1
PDGF	Platelet-derived growth factor
PF	Pulmonary fibroblasts
SD	Standard deviation
SEM	Standard error of mean
SM22	Transgelin
sm-MHC	Smooth muscle myosin heavy chain
sm-MLCK	Smooth muscle myosin light chain kinase
SNEA	Severe non-allergic eosinophilic asthma
TGF-*β*	Transforming growth factor *β*
TSLP	Thymic stromal lymphopoietin
