# Supplementary material for: Asthmatic Eosinophils Promote Contractility and Migration of Airway Smooth Muscle Cells and Pulmonary Fibroblasts In Vitro
Source: Cells. 2021 Jun 4;10(6):1389. doi: 10.3390/cells10061389 (PMC8229663; doi:10.3390/cells10061389)
Supplement: Supplementary file 1 [file cells-10-01389-s001.zip › cells-1182971-supplementary.pdf]

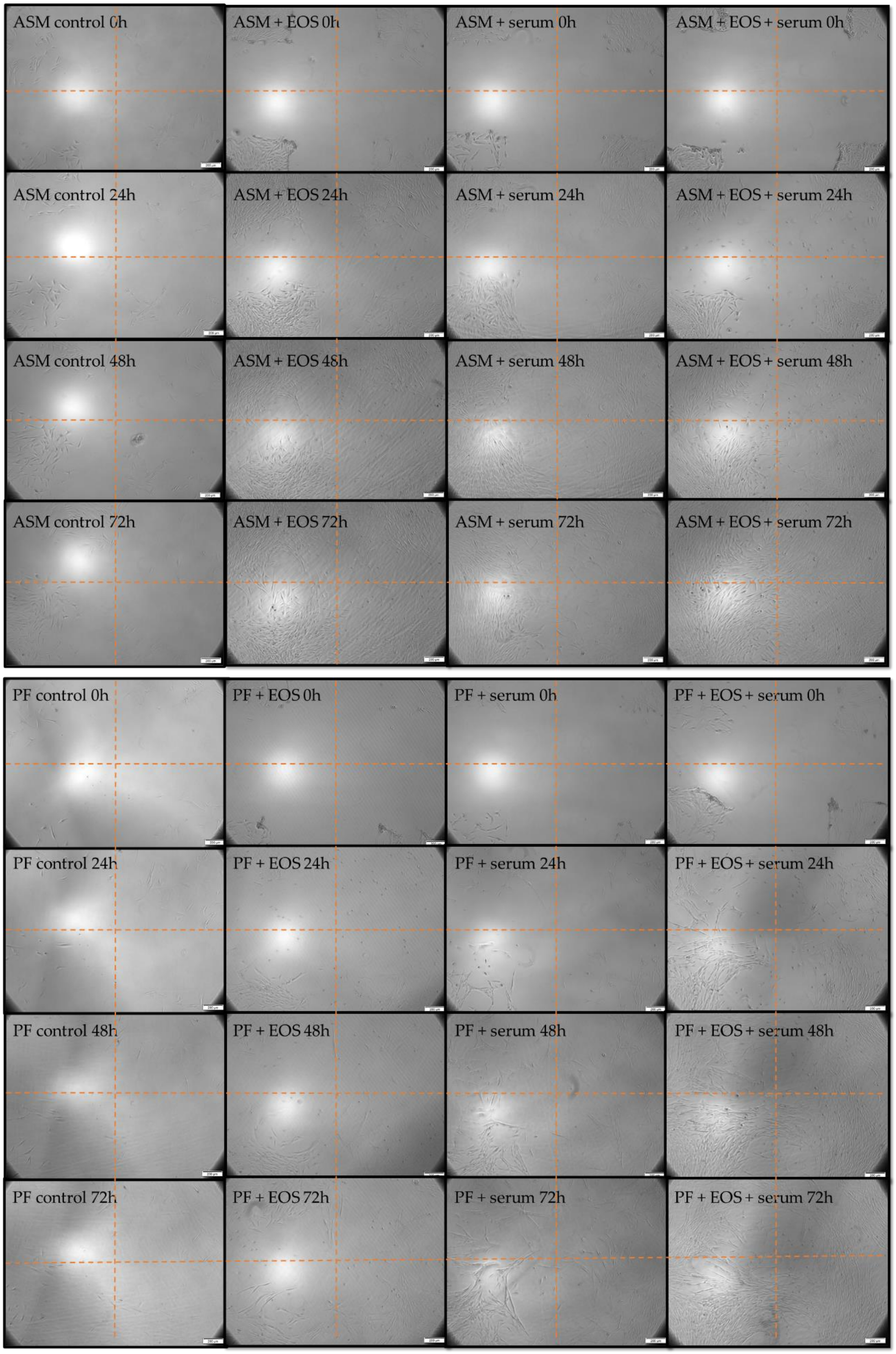

Figure S1. Images of ASMC and PF at 0, 24, 48, and 72 h time points. ASM – airway smooth muscle cells; EOS – eosinophil; PF – pulmonary fibroblasts.
